# Laser ablation-based techniques for microplastic analysis: recent advances and applications

**DOI:** 10.1039/d5ja00141b

**Published:** 2025-08-21

**Authors:** Pavlína Modlitbová, Lukas Brunnbauer, Gabriela Kalčíková, Aida Fazlić, Andreas Limbeck, Pavel Pořízka, Jozef Kaiser

**Affiliations:** a Central European Institute of Technology (CEITEC) Brno University of Technology Purkyňova 656/123 61200 Brno Czech Republic pavel.porizka@ceitec.vutbr.cz; b Institute of Chemical Technologies and Analytics Getreidemarkt 9/164-I2AC, 1060 TU Wien Vienna Austria; c Faculty of Mechanical Engineering (FME), Brno University of Technology Technická 2896/2 616 69 Brno Czech Republic; d University of Ljubljana, Faculty of Chemistry and Chemical Technology 113 Večna pot SI-1000 Ljubljana Slovenia

## Abstract

Microplastics have emerged as significant environmental contaminants due to the increasing production of polymer-based products and their limited disposal options. The persistence, bioaccumulation potential, and ability of microplastics to adsorb and transport toxic contaminants pose a risk to ecosystems and human health. Consequently, precise detection, characterization, and visualization of microplastics in various matrices are of paramount importance. However, the inherent challenges of analysing particles across broad size ranges with diverse physicochemical properties call for advanced analytical methods. This review focuses on two promising laser ablation-based techniques: Laser-Induced Breakdown Spectroscopy (LIBS) and Laser Ablation Inductively Coupled Plasma Mass Spectrometry (LA-ICP-MS). Both methods have demonstrated their utility in spatially resolved analyses, enabling the elemental characterization of microplastics. The review systematically evaluates existing studies employing these techniques, highlighting their benefits, limitations, and potential applications. Furthermore, it emphasizes the complementary nature of LIBS and LA-ICP-MS, advocating their tandem use for a comprehensive analysis of microplastics. By addressing current gaps in microplastic environmental research, this review aims to propose novel methodologies that can help to advance the understanding of the environmental fate and impacts of microplastics, facilitating the development of effective mitigation strategies.

## Abstract 2

Microplastics have emerged as significant environmental contaminants due to the increasing production of polymer-based products and their limited disposal options.

## Graphical abstract

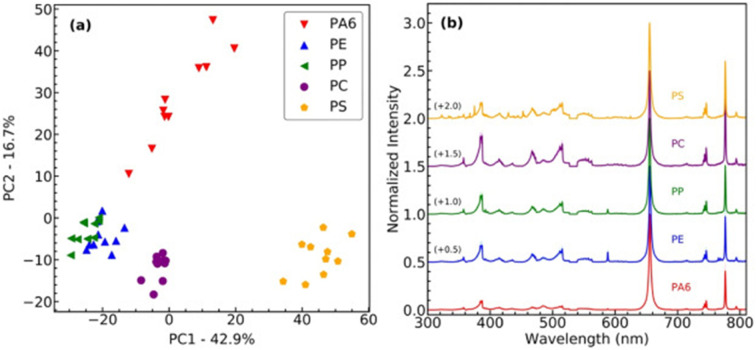

## 1. Introduction

Plastic pollution received significant attention in the past decade, primarily due to the increasing production of plastic and polymer products, coupled with their improper disposal and the consequent widespread environmental contamination.^1,2^ The issue extends beyond plastic items to small plastic fragments, such as microplastics (MPs), defined as particles ranging from 1 μm to 1000 μm.^3^ The environmentally relevant MPs research remains challenging because of the small particle size with wide distribution range, and because their size affects their physicochemical properties. Moreover, the MPs properties can easily be changed by surrounding environmental conditions such as sunlight irradiation, temperature variations, and presence of different organisms.^1,4^ Therefore, the consequent proper detection and characterization of MPs in various biotic and abiotic matrices are extremely challenging and time consuming, but of a paramount scientific interest.^2^

In general, detailed information of the MPs fate and behaviour in environmental samples and different matrices, especially in biota, is not investigated yet due to the vast complexity of processes involved. Thus, a demand arises for a novel analytical tool able to determine precise information on the spatial distribution of MPs in organisms together with their proper characterization. The requirement on the technology and instrumentation is strengthened by the necessity for large-scale imaging of whole-model organisms or their selected parts. Several techniques have been utilized in recent years to detect MPs in various environments as was well summarized in many reviews.^2,4,5^ Visual inspection methods (optical microscopy, stereomicroscopy), thermal analysis, chromatography, vibration-rotation spectroscopy techniques (such as Raman spectroscopy or Fourier-transform infrared spectroscopy, FTIR) and X-ray-based methods (such as scanning electron microscopy with energy dispersive X-ray spectroscopy, SEM-EDX) are typically used. However, inherently each technique comes with certain advantages and drawbacks limiting comprehensive analysis of MPs. Therefore, to fully characterize MPs, new analytical techniques need to be introduced and combined with existing approaches followed by the development of novel robust analytical tools.

Therefore, in response to current research needs, we aim to introduce and summarize the recent progress that has advanced significantly over the past few years in MPs analysis using two laser ablation-based techniques: laser-induced breakdown spectroscopy (LIBS) and laser ablation inductively coupled plasma mass spectrometry (LA-ICP-MS). The LIBS technique is well established in the spatially resolved elemental analysis of various matrices, including biotic ones, as well as in the analysis of polymers/plastics.^6–11^ Thus, the engagement of LIBS in the pioneering MPs studies was the natural progress.^12^ While LA-ICP-MS is a technique with a high spatial resolution, widely used for elemental analysis, especially in the biotic matrices,^13,14^ and geology,^15,16^ there are only few applications of LA-ICP-MS for the analysis of polymers/plastics.^17–21^ LA-ICP-MS seems to be very suitable thanks to the advantageous elemental analysis of polymers. This can be done by two approaches (i) by the detection of elements typically present as additives in polymers which differ in individual polymer types, thus, can easily serve as markers to distinguish different polymers^22^ or (ii) by monitoring the adsorption and desorption (leaching) of metals which are present as contaminants.^23^ For both techniques, an advanced statistical data analysis is currently widely used in spectral data processing and sorting.^24,25^ As a result, sample characterization and classification has advanced in last years. Both techniques started to be used in MPs analysis around 2020, and all existing studies will be introduced chronologically in this review together with the discussion about their benefits and drawbacks.

To very briefly introduce both methods, LIBS and LA-ICP-MS use the laser ablation as a sampling technique prior to the measurement. LA-ICP-MS offers a great sensitivity (ng g^−1^) and a fine spatial resolution (single digit μm), however, a very high acquisition and operating costs make this technique less accessible.^13,26^ On the contrary, the LIBS technique has a good sensitivity (μg g^−1^) and a satisfactory spatial resolution (25–100 μm), but both the speed and price of the analysis differ by two orders of magnitude with respect to LA-ICP-MS, with no request for a special atmosphere or vacuum.^6,27^ Also, both techniques are capable of simultaneously analysing various elements of interest, nutrients or other contaminants, so even their presence or a possible translocation can be established during the same analysis by this multi-elemental approach.^28^ To sum up, both offer distinct advantages for spatially resolved MPs analysis across various matrices, and the most appropriate technique should be selected according to purpose of analysis, available time and budget, and sample number and size. Moreover, both techniques can be beneficially used in tandem^29^ and the obtained results complement each other appropriately as shown many times before, even in the MPs research.

In summary, this review presents two newly adopted techniques that appear highly suitable and promising for the investigation of MPs in environmental contexts. While both techniques are capable of analysing samples across all three states of matter, their application is particularly advantageous for spatially resolved analyses of solid samples. All relevant studies addressing these methodologies are thoroughly introduced, systematically catalogued, and described in detail. Additionally, this review identifies key research gaps, highlights future directions, and critically evaluates potential solutions to advance the field of MPs research.

## 2. Microplastics in the environment

MPs enter the environment through various sources and pathways, including wastewater discharge, stormwater runoff, atmospheric deposition, and improper waste disposal. Once released, they accumulate in diverse ecosystems, such as freshwater bodies, oceans, soil, and even remote regions like polar ice and deep-sea sediments.^30–33^ When they are present in the environment, they undergo significant modifications due various aging processes. These interactions may lead to profound changes in their properties, which could affect their behaviour and impact in ecosystems.^34^ The processes that affect MPs are divided into biotic and abiotic.^35^ The most important abiotic processes include hydrolysis, thermal degradation, mechanical abrasion and oxidation by UV radiation.^36^ They can all lead to changes in the surface of MPs and the formation of new functional groups on the polymer surface that increase reactivity or promote further degradation.^37–39^ At the same time, biotic aging occurs when microorganisms attach to the surface of microplastic particles and form biofilms.^40,41^ This biofilm is composed of different microorganisms encased within a matrix of extracellular polymeric substances. This layer might completely cover the particles, which leads to further changes in the surface composition and properties.^37,42^

During their lifetime in the environment and as they undergo aging processes, MPs interact with a wide range of pollutants. These interactions frequently result in the adsorption and accumulation of metals, organic contaminants (such as persistent organic pollutants and polycyclic aromatic hydrocarbons), and nutrients. Factors such as the surface chemistry of MPs, environmental conditions, and the presence of biofilms can further influence the extent and nature of these interactions. As a result, MPs can act as carriers, facilitating the transport of hazardous substances across different ecosystems and potentially increasing their bioavailability to organisms.^43–45^

## 3. Conventional approaches

Until now, several techniques have been utilized to detect MPs in various environments as summarized in many comprehensive reviews.^2,4,5^ Therefore, very briefly, methods used in MPs research are typically divided into two groups, techniques utilized for the MPs identification or for their quantification.^4^ Mechanical-based techniques (*e.g.*, Tensile Tests, Lorentz Contact Resonance Imaging, Atomic Force Microscopy, AFM), thermal-based techniques (*e.g.*, Thermogravimetric Analysis, Differential Scanning Colorimetry, Nano-Localized Thermal Analysis, Pyrolysis-Gas Chromatography-Mass Spectrometry, Pyr-GC/MS, Thermal Extraction and Desorption Gas Chromatography-Mass Spectrometry, TED-GC/MS), spectral-based vibration-rotation techniques (*e.g.*, FTIR, Raman spectroscopy), spectral-based fluorescence techniques (*e.g.*, fluorescence lifetime imaging microscopy, FLIM), or spectral emission-based techniques (*e.g.*, LIBS) are used most often for the identification of MPs. Whereas for the quantification of MPs, microscopy-based techniques (*e.g.*, optical and electron microscopy, fluorescence microscopy, microscopy imaging) and mass-spectrometry-based methods (*e.g.*, Pyr-GC/MS, TED-GC/MS, Liquid Chromatography with tandem mass spectrometry, LC-MS/MS, matrix assisted laser desorption ionization with time of flight mass spectrometry, MALDI-TOF MS) are used. All these techniques offer various benefits as well as disadvantages, nevertheless they are irreplaceable in MPs analysis. Detailed information about these techniques has been objectively summarised by Shi *et al.* (2024) in the recent comprehensive review.^4^ In line with their literature research, FTIR and Raman spectroscopy are still the most used in the MPs analysis because of the existing spectral libraries for various polymer/plastics, even the degraded ones, and due to their rapid and very sensitive chemical analysis of MPs in complex environmental matrices.

The quantification of MPs still remains very problematic due to many variables that describe the properties of MPs, *e.g.*, size, shape, colour, chemical composition, and due to differences in sampled environmental matrices. For different states of matter, very different physical and chemical properties are important, *e.g.*, grain size distribution for sediment samples. This topic deserves a lot of scientific attention^46^ and many recommendations need to be kept for a proper inter-study comparability. These recommendations are based on how reliably a parameter is measured, and on the parameter's importance for the comparability. The geometric and the statistical means, *e.g.*, the multidimensional vector approach, where one vector contains the information for MPs distribution in one sample,^46^ can be used to quantitatively compare different studies.

## 4. Methods

The review of research papers was done on January 1st 2025 through Web of Science. Firstly, by using the ≫microplastic (All Fields) ≪ and ≫ LIBS (All Fields) ≪ keywords for the topic with ≫ article ≪ selected as the document type, 16 articles were identified with these search criteria. Then only 11 papers were manually selected as directly relevant to the topic of MPs analysis by LIBS. Secondly, by using the ≫ microplastic (All Fields) ≪, ≫ Laser Ablation (All Fields) ≪ and ≫ LA-ICP-MS (All Fields) ≪ keywords for the topic with ≫ article ≪ selected as the document type, 6 articles were identified with these search criteria. Google Scholar was then used to search for the latest research papers, and two recent papers were found and added manually.

## 5. Innovative laser-based techniques

### 5.1. Laser-induced breakdown spectroscopy

The LIBS technique is well established in the field of spatially resolved elemental analysis of various samples, including biotic, geologic, industry, or polymer/plastic.^6,7,10,11,47^ This technique can achieve good sensitivity (down to units of ppm) and satisfactory lateral resolution (down to 25 μm) at a high speed (up to 100 Hz laser pulse frequency) and acceptable analysis cost, with little to no requirements for a special atmosphere or vacuum, enabling measurements directly in air, including remote or *in situ* applications.^6,27^ Also, the multielement analysis capability is a great advantage, *i.e.*, the presence or the spatial distribution of various elements of interest, such as selected nutrients or artificially added contaminants can be monitored at the same time.^6^ Despite the significant advancements in this technique over the past decades, several limitations still persist. The most challenging issues include limited sensitivity, which may be insufficient for certain applications, and difficulties in obtaining quantitative results due to strong matrix effects and a limited number of matrix-matched standards.

The basic principle and instrumentation as well as the recent progress are described in detail elsewhere.^48–51^ Very briefly, LIBS is an optical emission analytical technique able to analyse all three states of matter with a capability for spatially resolved analysis of solid samples (depth profiling, 2D mapping). The relatively simple and robust LIBS set-up typically consists of a laser source (the most common are nano- and femtosecond solid phase pulsed lasers), focusing and collection optics components (*e.g.*, singlet, aspherical lens, microfocusing objectives), an optical fibre, a spectrometer (typically in Czerny–Turner or Echelle arrangements), and a detector (*e.g.*, intensified photodiode array, charge-coupled device, CCD, intensified CCD, ICCD, electron-multiplying CCD, EMCCD, complementary metal-oxide semiconductor, CMOS, or scientific CMOS, sCMOS).

In this chapter, research dealing with MPs analysis by LIBS were firstly summarized in Table 1 together with basic details about analysed matrices, types of MPs, used LIBS set-ups, experimental parameters, sample pre-treatments as well as used reference or complementary techniques. In Table 2, the detected elements or molecular bands are summarized together with the used emission wavelengths for each study separately. Of course, many other studies are more or less marginally touching the topic of MPs analysis by LIBS, *e.g.*, the papers by prof. Koch research group are dealing with depth profiling of artificially aged PE, PP, and PS plastics.^52,53^ Such papers will be in another section of this review, nevertheless, they are already discussed in more relevant reviews.^4,54–56^

**Table 1:** Chronologically ordered list of research LIBS MPs studies with a basic description of experiments^a^

Plastics/MPs, matrix (M), substrate (S)	Plastics/MPs characterization (size, shape)	Sample treatment	Instrumentation	Reference and complementary methods	Aims of the work	Ref.
Pristine MPs: PPEnvironmental MPs: PP, PS, PE, PC, PET, nylonM: seawaterS: polyethylene film	Pristine MPs PP ≈ 150 μmEnvironmental MPs > 3 μm	Pristine MPs PP: treated with Pb^2+^ and Cd^2+^ solution, suspend solutions filtered, air-driedEnvironmental MPs: seawater filtered through glass fiber, treated with H_2_O_2_, air-dried	SP LIBSNd:YAG laser(1064 nm)50 mJ per pulseGD (—), GW (—)	Raman spectroscopyOptical microscopy	- Single particle analysis- Detection of heavy metals- Quantitative analysis	57
Environmental MPs: PA, PE, PP, PVC, PET, PC, PSM: river sedimentsS: white or transparent solid rectangular plates	Environmental MPs > 500 μm	MPs extractions separated with NaCl solution, treated with H_2_O_2_ + Fe^2+^ solution	SP LIBSNd:YAG laser(1064 nm)6.44 mJ per pulseGD (0.2 μs), GW (1 μs)	FTIRStereomicroscopyOptical microscopy	- MPs classification, PCA- MPs distinguishing among non-plastic (natural) microparticles	12
Pristine MPs: PEEnvironmental MPs: PE, PP, PS, PET, PMMA, rayonM: beach sedimentS: —	Pristine MPs PE: 2–3 mm, flakeEnvironmental MPs: 0.5–5 mm; flake, fragment, fiber, granule, film, foamed	Oven-dried at room temperature	SP LIBSNd:YAG laser(1064 nm)50 mJ per pulseGD (—), GW (—)	FTIR	- Detection of heavy metals- Semiquantitative analysis	58
Pristine MPs: HDPE, PBAT, PET, PLA, PP, PS, PVC, nylonEnvironmental MPs: PE, PPM: marine beach sandS: —	Environmental MPs: 2–5 mm; cylinder, disk, masterbatch, sphere	Environmental MPs: filtered with a Giuliani sieve, washed, dried	DP LIBSNd:YAG laser(1064 nm)30 mJ per pulseGD (1 μs), GW (2000 μs), ID (1 μS)	Optical microscopy	- Detection of heavy metals- MPs classification, PCA	59
Pristine plastics: PET, PAEnvironmental MPs: no characterizationM: stream waterS: dried 50 μL drops on glass slides	Pristine plastic PET: 15 × 15 × 0.3 mmPristine plastic PA: 12 × 3.5 mm; circleEnvironmental MPs: 45–1000 μm	Pristine plastics: immersed in distilled/stream water, part treated with Cu^2+^ solutionEnvironmental MPs: filtered through mesh steel filters, part of them treated with H_2_O_2_ solution, then part of them treated with Cu^2+^ solution	SP LIBSNd:YAG laser(1064 nm)100 mJ per pulseGD (8 μs), GW (0.3 μs)	—	- Cu detection in MPs/plastic waste- Detection of heavy metals	60
Pristine MPs: PA, PE, PET, PP, PVCM: freshwater/wastewater agedS: fixed onto epoxy resin	Pristine MPs PE: 116.3 ± 68.0 μmPristine MPs PP: 357.0 ± 46.7 μmPristine MPs PA: 455.0 ± 49.1 μmPristine MPs PET: 23.1 ± 7.2 μmPristine MPs PVC: 157.4 ± 127.7 μmAll pristine MPs: fragments	Pristine plastics were grounded in a centrifugal mill to prepare MPs, part of MPs treated with freshwater/wastewater, filtered	SP LIBSNd:YAG laser(532 nm)5 mJ per pulseGD (500 μs), GW (50 μs)	Optical microscopySEMLaser diffraction analysisLA-ICP-MSICP-MSRaman spectroscopy	- Direct analysis of MPs with developed biofilm- Characterization of MPs, PCA	22
Pristine plastics: Teflon, PP, PEEnvironmental MPs: PE, PP, PETM: estuary waterS: dried 10 μL drops on a Teflon slide	Pristine plastics PP, PE: 1 cmEnvironmental MPs: 1–5 mm	Pristine PE, PP: treated with Pb^2+^ solution, wiped with neat and soft tissue paperEnvironmental MPs: Water samples were sieved, dried, treated with H_2_O_2_ + Fe^2+^ solution, separated by using NaCl solution	SP LIBSNd:YAG laser(532 nm)4/10 mJ per pulseGD (—), GW (—)	Raman spectroscopy ICP-OES	- Integrated LIBS-Raman system- Detection of heavy metals and trace elements	61
Pristine plastics: PADCEnvironmental MPs: no characterizationM: seawaterS: mounted on a rotating stage	—	Environmental and pristine MPs: manually cleaned with deionized water	SP LIBSNd:YAG laser(1064 nm)55 mJ per pulseGD (—), GW (—)Ar atmosphere	FTIR	- LIBS + CF LIBS for MPs classification- Quantitative (C, O) MPs analysis	62
Pristine MPs: PP, PTFE, PVC, PC, PET, PS, PPM: —S: mounted in acrylic resin	Pristine MPs ∼ 5–270 μm	Pristine plastics were grounded in a centrifugal mill to prepare MPs, MPs embedded to silicon wafers with acrylic resin, cross-sectioned by manual polishing	SP LIBSArF laser(193 nm)GD (0.1 μs), GW (30 μs)He, Ar atmosphere	LA-ICP-MSICP-MSOptical microscopy	- Simultaneous LIBS/LA-ICP-MS- Characterization of MPs- Fluorine detection (laterally resolved)- Quantitative analysis of elements in MPs (laterally resolved)	29
Environmental MPs: PE, PP, PETM: estuary river waterS: —	Environmental MPs: 1–5 mm; fragments, films, and fibers	Water sieved with screens, dried, treated with H_2_O_2_ + Fe^2+^ density separation with ZnCl_2_ solution	SP LIBSNd:YAG laser(532 nm)4 mJ per pulseGD (0.7 μs), GW (10 μs)	FTIRRaman microscopySEM-EDSOptical microscopy	- Integrated LIBS-Raman system for MPs characterization- Detection of heavy metals	63
Pristine MPs: PEM: human tonsilsS: epoxy embedded onto glass slide	Pristine MPs: PE: 1–4 μm, fragments; PE: 3–16 μm, spheres	Human tonsils were spiked with PE MPs, digested, filtered, residua collected	SP LIBSNd:YAG laser(532 nm)40 mJ per pulseGD (0.5 μs), GW (50 μs)	Raman spectroscopyXRFFTIRLaser diffraction analysisFE-SEM	- Introducing optimized protocol for MPs detection in human soft tissue- Characterization of MPs, PCA	64

a — Data not presented; PA (polyamide), PADC (polyallyl diglycol carbonate), PBAT (polybutylene adipate terephthalate), PC (polycarbonate), PE (polyethylene), PET (polyethylene terephthalate), PLA (polylactic acid), PP (polypropylene), PS (polystyrene), PTFE (polytetrafluoroethylene), PVC (polyvinyl chloride), PMMA (polymethyl methacrylate), XRF (X-ray fluorescence), SEM-EDS (scanning electron microscopy energy dispersive X-ray spectroscopy), FE-SEM (field emission scanning electron microscopy).

**Table 2:** Chronologically ordered list of research LIBS MPs studies with information about the investigated elements and their emission lines in LIBS experiments

Plastic/MPs type	Element (emission line)	Ref.
Pristine and environmental MPs	Al (237.2 nm), Hg (364.9 nm), Cd (226.4 nm, 643.9 nm), Pb (239.4 nm, 368.4 nm), C (247.9 nm), Cr (428.9 nm)	57
Environmental MPs	CN band (383–387 nm), C_2_ band (458–475 nm, 495–518 nm, 540–563 nm), CH band (480–491 nm)	12
Pristine and environmental MPs	C (247.9 nm), Mn (213.65 nm, 403.3 nm), Fe (259.2 nm), Pb (405.8 nm), Cu (324.3 nm), Zn (213.7 nm), Cd (360.4 nm), Cr (430.2 nm)	58
Pristine and environmental MPs	C (247.9 nm), CN band (370–390 nm), H (656.3 nm), Al (394.4 nm, 396.2 nm), Fe (373.5 nm, 373.7 nm), Pb (405.8 nm), Cr (425.4 nm, 427.5 nm)	59
Pristine plastics and environmental MPs	Cu (324.75 nm)	60
Pristine MPs	K (766.49 nm, 769.89 nm), CN band (≈388 nm), Ca (385–400 nm), Na (589 nm), H (656.28 nm)	22
Pristine plastics	Pb (405.83 nm), CN band (388.29 nm)	61
Environmental MPs	Mg (517.3 nm, 518.4 nm), Li (615.5 nm, 670.8 nm), Al (308.21 nm, 309.28 nm, 394.38 nm, 396.18 nm), Mn (344.24 nm, 346.04 nm, 347.45 nm, 432.66 nm), Ni (300.25 nm, 305.15 nm, 310.20 nm, 336.16 nm, 337.42 nm, 471.63 nm), Zn (328.24 nm, 330.26 nm, 334.50 nm, 472.21 nm), Cr (357.87 nm, 359.37 nm, 360.52 nm, 404.05 nm, 405.92 nm, 406.79 nm, 425.44 nm, 427.44 nm, 428.99 nm), Cu (324.76 nm, 327.41 nm, 427.70 nm), Ca (643.9 nm, 646.3 nm, 393.97 nm, 396.85 nm, 422.79 nm), Pb (405.83 nm), CN band (388.29 nm)	
Pristine plastics and environmental MPs	C (492.7 nm, 815.7 nm, 865.6 nm, 267.0 nm, 313.0 nm, 838.1 nm), O (405.7 nm, 422.4 nm, 452.4 nm, 851.0 nm, 345.6 nm, 408.1 nm, 417.0 nm, 485.7 nm)	62
Pristine MPs	F (685.6 nm, 684.72–686.4 nm, 738.75–741.08 nm, 774.25–776.02 nm), C (248 nm, 247.45–248.29 nm, 832.12–834.91 nm, 939.01–941.63 nm), Cl (837 nm, 836.56–838.50 nm), C_2_ band (515 nm, 515.83–516.73 nm), O (777 nm, 776.13–778.70 nm, 842.97–845.50 nm), H (649.07–664.53 nm), CN band (386.69–388.84 nm)	29
Environmental MPs	Ca (373.68, 393.41, 428.45 nm), Mg (333.62, 383.81, 389.44 nm), Al (394.39, 396.19 nm), Co (343.3, 347.43 nm), Ni (323.34, 336.92, 356.65 nm), Zn (330.25, 334.49 nm)	63
Pristine MPs	C (247.88 nm), Ca (393.0 nm, 396.88 nm), CN band (358.1 nm, 388.34 nm), Mg (279.55, 280.27)	64

#### 5.1.1. Sample preparation

It is widely accepted within the LIBS community that sample preparation is unnecessary prior to LIBS analysis. This is true only for several cases (*e.g.*, specific *in situ* or remote analysis, specific industry or geological applications).^65–67^ However, environmental samples containing MPs usually need to be thoroughly prepared to eliminate observational errors arising from the incorrect collection, sample handling, storage, transport, or final preparation before the LIBS analysis of the samples.

The investigated environmental matrices containing MPs were typically aquatic (seawater, estuary, or stream water) or solid (river sediments, beach sediments, or marine beach sands). Then, the laboratory prepared *e.g.*, ground in centrifugal mill^29^ MPs and/or artificially aged or artificially metal-contaminated MPs were subjects under the investigation.

The environmental aquatic or solid samples were usually transported into the laboratory in steel/glass containers for an appropriate number of replicates and a sufficient volume/mass. The aquatic samples were filtered through glass fibres, treated with Fenton's reagents (H_2_O_2_ and Fe^2+^), which removes organic matters in typical conditions, *i.e.*, temperature set at 65 °C, samples shaken at 80 rpm for no more than 72 hours.^68^ Then the digest was filtered and placed in a glass Petri dish and air-dried overnight. The sediment or sand samples were mostly only filtered with a Giuliani sieve, washed, and air dried.^59^ Then single MPs were picked using tweezers and placed on a suitable substrate for LIBS and other analyses. Also, the sediments could be separated from the rest of matrices by density separation – this procedure is commonly used in MPs analysis and well described elsewhere.^69^ Typically, environmental samples are mixed with saturated NaCl or ZnCl_2_ solutions for several hours, for example in a MicroPlastic Sediment Separator.^12^

Then, the MPs have to be fixed into appropriate substrates which are not interfering with LIBS analysis. Most often, MPs were fixed into epoxy^22^ or acrylic resin,^29^ onto glass slides, mounted directly on a rotating stage,^62^ or in the case of aquatic MPs suspensions also applied onto various substrates as glass^60^ or Teflon^61^ slides in the form of dried droplets. Each fixation process has its benefits as well as disadvantages. Minimal interference of the substrate with the tested MPs, a stable fixation which guarantees that the MPs will be strongly attached and will not fall out or be removed from the substrate during the laser beam interaction, and of course, the simplicity, the speed, the price, and the availability of the preparation procedures are the most important factors.

Uniquely, a biotic matrix – human tonsils – was artificially spiked by PE MPs (fragments and spheres) and then the tonsils were alkaline digested, filtered, the MPs residua were collected and analysed after fixation into epoxy resin.^64^ This work outruns others because it is the first evidence of a LIBS analysis of human matrix which significantly suggests a possible usage of this technique in medical MPs research, even though the introduced sample preparation protocol for MPs detection in human soft tissue does not allow for a spatially resolved MPs analysis.

Pristine and environmental MPs various in shapes (flake, fragment, fibre, granule, film, foamed, cylinder, disk, masterbatch, or sphere), sizes (ranging from 1 to 1000 μm), or types (PBAT, PLA, PVC, PP, PS, PE, PC, PET, PA, PVC, rayon, acrylic, nylon, and Teflon) were included in the reviewed studies. The overview of the measured MPs is listed in the following Table 3 together with the information about their polymer type, lattice structure, chemical formula, chemical structure, C/H ratio, and usage in common life.

**Table 3:** The listed analysed MPs with basic information about their properties and usages. Based on ref. 10 and 70a

MPs type	Polymer	Lattice structure	Chemical formula	Chemical structure	C/H ratio	Usage
PA (≈nylon)	Polyamide	Semi-crystalline	(C_6_H_11_NO)_*n*_	Aliphatic	0.545	Synthetic fibers, construction materials
PADC (=CR-39)	Polyallyl diglycol carbonate	—	(C_12_H_18_O_7_)_*n*_	Aliphatic	0.667	Optics
PBAT	Polybutylene adipate terephthalate	Semi-crystalline	(C_20_H_30_O_10_)_*n*_	Aromatic	0.667	Bags and wraps
PC	Polycarbonate	Amorphous	(C_16_H_14_O_3_)_*n*_	Aromatic	1.143	Lenses, screens, safety glasses
PE	Polyethylene	Semi-crystalline	(C_2_H_4_)_*n*_	Aliphatic	0.5	Bottles, films, sheets
PET	Polyethylene terephthalate	Amorphous/semi-crystalline	(C_10_H_8_O_4_)_*n*_	Aromatic	1.25	Bottles, food containers, clothes
PLA	Polylactic acid	Amorphous/semi-crystalline	(C_3_H_4_O_2_)_*n*_	Aliphatic	0.75	Disposable tableware, cutlery, electronics
PP	Polypropylene	Semi-crystalline	(C_3_H_6_)_*n*_	Aliphatic	0.5	Sterilizable hospital, equipment, toys
PS	Polystyrene	Amorphous	(C_8_H_8_)_*n*_	Aromatic	1	Insulation material
PTFE (≈Teflon)	Polytetrafluoroethylene	Semi-crystalline	(C_2_F_4_)_*n*_	Aliphatic	—	Insulation of wiring, slide plates
PVC	Polyvinyl chloride	Amorphous	(C_2_H_3_Cl)_*n*_	Aliphatic	0.667	Water pipes, gramophone records, electric cables
PMMA (=acrylic, plexiglass)	Polymethyl methacrylate	Semi-crystalline	(C_5_H_8_O_2_)_*n*_	Aliphatic	0.625	Glasses, rear light, video disks, sheets
Rayon	Sodium cellulosate	—	(C_6_H_9_O_4_–ONa)_*n*_	Aromatic	0.667	Clothes

a — Data not available.

Moreover, not only pristine MPs but also pristine plastic of a bigger size, *e.g.* 1 cm in diameter^61^ were used for the optimization of experimental set-ups and parameters so followingly environmental or artificially aged/contaminated MPs could be analysed with the most possible sensitivity and accuracy.

#### 5.1.2. Experimental set-ups and data analysis

In the analysis of MPs, the most commonly used laser is the pulsed nanosecond solid phase Nd:YAG operating mainly at two wavelengths (1064 nm and 532 nm) with pulse energies starting from 4 mJ up to 100 mJ per pulse.^60,61^ Only once, the excimer laser operating at 193 nm was used.^29^ Both the single pulse (SP) and the double pulse (DP) arrangements were used. Surprisingly, DP-LIBS was employed only in one case^59^ despite the immense advantages of this setup including the increase of the sensitivity of the LIBS measurements. The single-particle analysis^57^ was also successfully employed despite the fact that this type of experiment is very time-consuming due to the necessity of a manual focusing of the laser beam on each particle separately.

Most commonly, the experiments were set in laboratory conditions and in an air atmosphere. Nevertheless, also the Ar and He atmosphere were reported to be helpful and suitable for MPs analysis.^29,62^ The quantitative,^57^ semi-quantitative^58^ as well as qualitative^59^ analyses were employed in specific cases of various elemental content analysis. The CF (calibration free) LIBS was once introduced for MPs classification as well as for quantitative analysis of C and O in PADC and in non-specified environmental MPs.^62^ The identification of the MPs type or the differentiation between variously artificially aged MPs were done by an advanced multivariate data analysis. The principal component analysis (PCA) was typically employed for a dimensionality reduction and for a useful visualization. Subsequently, the clustering or classification was done by yet another algorithm.^22,64^ The processing through PCA-based is rather straightforward task given typically lower number of samples (given number of investigated polymers). Hence, the field of MPs is still underdeveloped in terms of more complex and extensive clustering and classification. This might be the scope of future works focused on the fate of MPs in the environment when dealing with various aging mechanisms which will interfere with the signal response.

The LIBS became a well-established standalone method over the times. However, the increasing demands on the accuracy and the correctness of measurements require utilization of other techniques. This could be done by two different approaches. Either another technique could be used in tandem with LIBS as, *e.g.*, simultaneous detection with LA-ICP-MS^29^ or the integrated LIBS-Raman spectroscopy system.^63^ The second and the most common approach is using other techniques separately step by step. For example, LIBS data could be a suitably supplemented by results from optical microscopy,^57^ stereomicroscopy,^12^ ICP-MS,^29^ FTIR,^62^ ICP-OES,^61^ SEM/SEM-EDS/FE-SEM,^22,63,64^ laser diffraction analysis,^22^ or XRF.^64^ In this case, the MPs could be prepared for each analysis in different and in the most appropriate way.

#### 5.1.3. Applications

##### 5.1.3.1. Identification of microplastic types

**Fig. 1:**
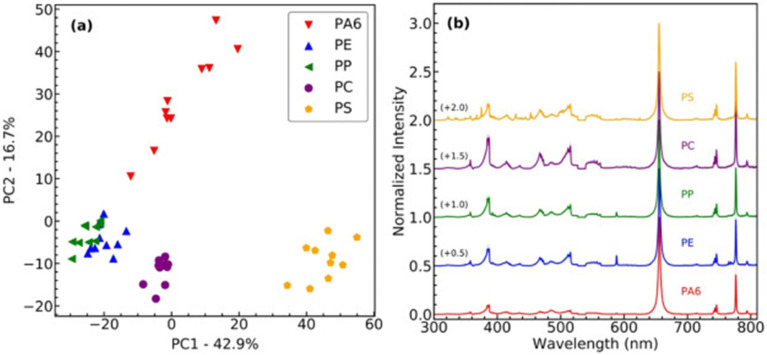
Plastic type identification for PS, PC, PA6, PE, and PP. (a) shows a PCA with the first two principal components separating the plastic types into four clusters. (b) shows the reference average MPs spectra. Reproduced from ref. 12 with permission from Elsevier copyright [2025].

The LIBS technique has shown to be a powerful technique for distinguishing types of MPs based on their unique emission spectra, selected spectral parts, or only emission lines. Studies have shown that LIBS can differentiate many types of MPs, such as PE, PP, and PS, by analysing their carbon, hydrogen, and oxygen emission lines. For example, LIBS has been used to classify MPs extracted from environmental samples, achieving high accuracy in distinguishing among polymers, even in complex matrices.^57^ In addition, the incorporation of chemometric techniques such as PCA has significantly enhanced the discriminatory power of LIBS for polymer identification. PCA enables the reduction of high-dimensional spectral data into principal components, allowing for a clearer visualization and classification of different polymer types. For instance, PCA has been successfully applied to LIBS spectra to differentiate MPs based on subtle variations in their elemental composition, even among chemically similar polymers as visible at Fig. 1.^12,59^ Furthermore, the combination of LIBS with advanced data processing algorithms has demonstrated a high efficiency in distinguishing differentially aged MPs (biotic *versus* abiotic aging process), which exhibit altered chemical profiles due to the environmental degradation.^22^

##### 5.1.3.2. Detection of heavy metals and trace elements

MPs often act as carriers for heavy metals and trace elements, either through adsorption from the environment or incorporation during manufacturing. LIBS is particularly effective in qualitative and quantitative analysis of these elements due to its sensitivity and multi-elemental analysis capability, Table 2. The detection of elemental lines (both atomic and ionic) is present together with molecular bands (CN and C_2_) that could be correlated with ablated polymer matrices. Obviously, the broad spectral detection gives redundant spectral information (multiple lines representing the same element). This in turn might lead to potential spectral interferences between individual spectral lines and even with molecular bands. Thus, it is necessary to control data processing with respect to potential spectral interferences.

For instance, LIBS has been applied to identify toxic metals such as Pb, Cd, and Hg adsorbed onto MPs surfaces from marine environments.^61^ Then LIBS was used to analyse the complex interactions between MPs surfaces and metal ions in both marine and freshwater environments, revealing significant variations in adsorption behaviour depending on polymer type and environmental conditions.^58,60^ Advanced chemometric tools, including Partial Least Squares Regression (PLSR) and PCA, have been employed to enhance the accuracy of heavy metal semi-quantification, making LIBS a robust tool for environmental monitoring of metal contamination on MPs. However, successful quantitative heavy metal analysis has only been reported for Pb and Cd.^57^ In this study, selected PP MPs were immersed in solutions of lead acetate and cadmium sulphate at five different concentrations for 24 hours. The contaminated particles were subsequently dried and analysed using single-particle LIBS (50 particles per concentration). The obtained spectra were averaged and normalized to the C 247.9 nm emission line to minimize the effects of varying particle morphologies and fluctuations in laser pulse energy.

##### 5.1.3.3. Single-particle analysis

This approach involves the characterization of individual MPs particles, allowing researchers to assess their composition, morphology, and associated contaminants. Single-particle LIBS provides valuable data on particle-specific heterogeneity, which is critical for understanding the behaviour and fate of MPs in the environment. Recent advancements in LIBS instrumentation, such as high-resolution spectrometers and automated particle detection systems, have further enhanced the precision and throughput of single-particle analysis.^29^ Studies utilizing this approach have demonstrated its capability to identify polymer types and detect adsorbed heavy metals on individual particles, thereby contributing to a more comprehensive understanding of MPs pollution.^60,63^

### 5.2. Laser ablation-inductively coupled plasma-mass spectrometry (LA-ICP-MS)

LA-ICP-MS is a widely used analytical technique for measuring the elemental content of solid samples.^71^ It is based on firing a laser on the sample surface and ablating material, which forms an aerosol. If the laser ablation parameters are selected carefully, the composition of the aerosol represents the sample's composition. The so-generated aerosol is transported to the ICP-MS for element-specific detection.

LA-ICP-MS excels due to its high sensitivity, large dynamic range, and spatial resolution. Typically, the elemental content for major, minor, and trace constituents can be detected (with LoDs ranging from μg g^−1^ to ng g^−1^ depending on the element of interest). Besides analysing the bulk content of samples, LA-ICP-MS enables the investigation of the spatial distribution of elements within a sample by either imaging or depth profiling. In this case, spatial resolution in the low μm range and depth resolution in the (sub) μm range are reported.^15^ Due to its many advantages, it is a well-established technique routinely applied in fields such as bioimaging^14,72^ and geology.^15,16^

LA-ICP-MS is suitable for analysing all kinds of solid samples (including non-conductive samples). During the LA sampling, the sample is typically in a He-atmosphere, meaning that it is not exposed to high vacuum, making it easily applicable to biological and life science samples. Another advantage of ICP-MS detection is that isotopic information is accessible^73^ and can be used to study environmental processes or investigate the geographic origins of samples.

Limitations of LA-ICP-MS include the limited sensitivity for non-metals and the challenges to obtaining quantitative results due to matrix effects. Typically, matrix-matched standards must be applied for reliable quantitative analysis. In the case of polymer analysis, this means that prior to analysing the metal content of a sample, the polymer type must be identified, and corresponding matrix-matched standards must be applied.^29,74^ Quantitative analysis is often hampered due to the availability of the required reference materials. In this case, in-house prepared and characterized standards are often used.

The previously discussed advantages of LA-ICP-MS may also benefit emerging fields such as MPs analysis. LA-ICP-MS has already successfully been applied to analyse polymer samples for characterizing the metal content for major, minor, and trace elements.^20,74,75^

Even though LA-ICP-MS cannot detect some of the main constituents of MPs (H, N, O), its outstanding sensitivity for metals is beneficial for characterizing the metal content of MPs. Here, the main interest lies in investigating the potential changes in the elemental content during environmental aging. During aging processes, additives present in the particles can leach into the environment. Additionally, heavy metals may accumulate on the surface of MPs or in the developed biofilm or even diffuse into the particle, posing an additional risk. Nevertheless, before successfully applying LA-ICP-MS analysis to MPs, several challenges have to be considered:

· Quantification: LA-ICP-MS typically requires matrix-matched standards to obtain quantitative results due to matrix-effects. Depending on the analysed matrix, the interaction of the laser with the sample results in different amounts of material being ablated and a different sized particle aerosol being generated. This leads to variations in the transport efficiency to the ICP-MS and can also influence ionization efficiency in the ICP due to differences in vaporization. In the case of MPs analysis, this means that prior to analysing the metal content of a particle, the polymer type must be identified, and corresponding matrix-matched standards must be obtained and applied to obtain reliable quantitative results.^29,74^ This typically requires additional analysis with other instrumentation and techniques and poses challenges in terms of sample transfer. Another option is tandem LA-ICP-MS/LIBS which can be used for combined polymer type identification and quantitative trace metal analysis.^29^ Therefore, many studies report only qualitative results which hinder straightforward comparability between different works.

· Detecting carbon: detecting MPs based on their carbon signal is often challenging due to elevated ambient background and limited sensitivity. Additionally, carbon is known for its two-phase transport,^76,77^ which complicates the analysis. Nevertheless, in many cases individual polymer types contain specific metals originating from the manufacturing process (*e.g.*, Sb_2_O_3_) is used as a catalyst in PET manufacturing^78^ or inorganic elements are present due to additives^79^ which can be used as marker elements for the detection.

· Sample preparation: due to their small size and irregular shape, MPs are challenging to analyse with LA-ICP-MS, as typically, a flat sample surface is required to achieve reproducible results.

#### 5.2.1. Sample preparation

As discussed in the LIBS part, LA-ICP-MS is often described as a so-called direct-solid-sampling technique that does not need sample preparation. Nevertheless, this is often not the case for many applications, as a flat sample surface is typically required for conventional analysis. Compared to LIBS this is even more crucial as LA-ICP-MS is typically operated with smaller spot sizes enhancing the negative effect of sample roughness and surface irregularities. In geology or bioimaging, where LA-ICP-MS is routinely applied, sample preparation strategies have been developed and improved continuously over the past years. Nowadays, in the field of bioimaging, often thin cuts of the sample under investigation are prepared either by cutting thin sections directly from frozen tissue or by preparing thin sections from paraffin-embedded tissue.^13,80^ In materials science and geosciences, typically polished minerals or thin cuts are prepared for analysis.

Due to the irregular shapes and small size of MPs, some kind of sample preparation must be done to accomplish LA-ICP-MS analysis. Nevertheless, in the case of analysing larger MPs (>1 mm), direct-solid-sampling without sample preparation can be considered.^81^ Additionally, if the MPs under investigation are small enough (<20 μm), a novel sampling technique based on non-destructive LA can be applied after the distribution of the particles on a suitable substrate,^82–84^ enabling directly analysing the MPs under investigation. Besides these two direct-solid-sampling strategies with virtually no sample preparation, a sample preparation approach based on mounting the MPs in a resin and preparing cross-sections is reported in the literature.^22,29^ In this case, preparing a flat sample surface can is suitable for LA-ICP-MS analysis. Nevertheless, potential alterations of the elemental distribution within the individual particles due to leaching or alteration of the sample in general during the mounting process must be considered.

#### 5.2.2. Experimental setups

Instrumentation used in LA-ICP-MS is typically not as diverse as in LIBS. On the LA side, instrumentation can be differentiated on the laser's pulse duration (typically either femtosecond or nanosecond) and on the wavelength (typically 266 nm, 213 nm, or 193 nm).^85^ The most used lasers are nanosecond lasers due to their lower price. However, the advantage of femtosecond lasers is reducing thermal effects during the ablation process limiting fractionation of the analytes.^86,87^ Regarding the wavelength, typically, a shorter wavelength results in an aerosol with a smaller particle size distribution generated from the ablation process, which is favourable for transportation and atomization/ionization in the ICP. Additionally, ArF excimer lasers operating at 193 nm typically have a better stability and a more homogeneous beam profile which is favourable.

On the ICP-MS side, various types of instruments are available, differing in how the separation of *m*/*z* is carried out. Each type has certain advantages and limitations regarding sensitivity, resolution, and multielement capabilities.^88^

· Quadrupole-based instruments (ICP-Q-MS) are the most widely used instruments due to their lower cost and high sensitivity. ICP-Q-MS systems detect the selected *m*/*z* sequentially, limiting multielement detection capabilities with a limited mass resolution.

· Time-of-flight-based instruments (ICP-TOF-MS) have only recently been introduced. These instruments enable true simultaneous multielement detection with adequate mass resolution. Nevertheless, the sensitivity is limited compared to quadrupole and sector field-based systems.

· Multi collector-sector field ICP-MS (MC-ICP-SF-MS) are typically applied for high precision measurements of isotope ratios due to their outstanding mass resolution and sensitivity. Nevertheless, multielement capabilities are typically limited to a small number of selected *m*/*z*.

This is also reflected in the instrumentation used in the reviewed MPs papers: on the laser ablation side, either state-of-the-art nanosecond ArF excimer lasers operating at a wavelength of 193 nm were applied^22,82,83^ or frequency multiplied nanosecond Nd:YAG lasers operating either at 266 nm (ref. 89) or at 213 nm (ref. 81) were used. On the ICP-MS side, only quadrupole-based instruments are reported.

Nevertheless, recent developments in the available instrumentation for LA-ICP-MS may provide substantial benefits for MPs analysis, boosting its applications. Therefore, we would like to present and discuss these developments shortly: with the recent introduction of ICP-TOF-MS (time-of-flight) instruments, simultaneous multielement detection became available in LA-ICP-MS imaging, which is not possible with quadrupole-based instruments due to its sequential detection.^90^ With this type of instrument, LA-ICP-MS measurements may become even more relevant to investigate and understand the environmental fate of MPs. Many different elements may play a role in MPs' physical and chemical degradation processes, and the presence of certain metals may govern biofilm formation. The use of ICP-TOF-MS instruments in combination with LA enables non-targeted simultaneous multielement analysis, which is a vital tool for understanding environmental processes. Nevertheless, quadrupole-based instruments can still provide valuable insights into MPs due to their better sensitivity and wide availability but require preliminary selection of the analytes of interest.

On the laser ablation side, improvements in the ablation cell design (rapid response cells) have substantially reduced washout times, resulting in a better sensitivity and faster analysis.^91^ Additionally, these novel cells, combined with improvements in data acquisition rates of ICP-MS instruments, open new possibilities in terms of the fundamentals of LA-ICP-MS imaging.

#### 5.2.3. Measurement concepts

**Fig. 2:**
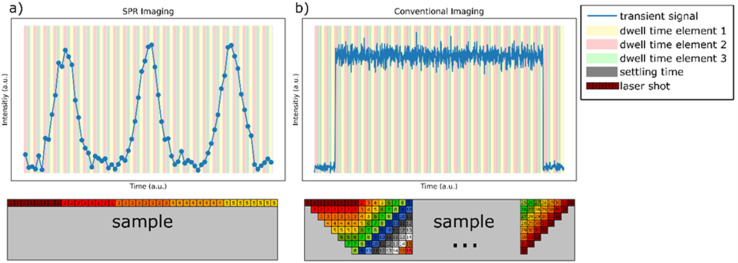
Difference in the two measurement modes used for LA-ICP-MS imaging: (a) single-pulse response (SPR) imaging and (b) conventional imaging of a homogeneous distribution of an analyte.

Conventionally, LA-ICP-MS imaging experiments are carried out in a so-called continuous scan mode.^92,93^ Using this approach, the signal from individual shots is not fully resolved resulting in a plateau-like signal with overlapping laser shots being fired (Fig. 2a). While this approach enables the generation of elemental maps, certain limitations must be considered: firing overlapping laser shots results in multiple shots being fired in the same position. Therefore, the homogeneity of the analysed sample under investigation must be assumed. Additionally, this approach introduced an inherent blur into the recorded images.

Besides the conventional continuous scan mode, single-pulse response (SPR) imaging has recently been proposed and applied to LA-ICP-TOF-MS imaging. In this case, the signal generated from each laser shot (and, therefore, each pixel) is fully resolved (Fig. 2b). This results in the best possible lateral resolution and only one laser shot fired per location. Especially the fact that no assumptions about the sample homogeneity in depth are necessary may prove beneficial considering analysing environmental MPs where no previous knowledge about the sample is available.

#### 5.2.4. Applications

Table 4 provides a chronological overview of applications of LA-ICP-MS in the field of MPs analysis. Recently, studies have emerged focusing on the direct analysis of nanoplastics (NPs), defined as particles ranging from 0.001 to 1 μm size.^3^ Given their significant contribution to environmentally relevant research, these studies are discussed alongside other key research works in the following paragraphs.

**Table 4:** Chronologically ordered list of research LA-ICP-MS MPs and NPs studies with a basic description of experiments^a^

Particles, matrix (M), substrate (S)	Characterization (size, shape, labels)	Sample treatment	Instrumentation	Reference and complementary methods	Aims of the work	Ref.
**Microplastics**						
Environmental MPs: PP, PEM: beach sedimentS: —	Environmental MPs: PP and PE in mm range	MPs were sieved at 5 and 1 mm, MPs selected by plastic tweezersPart of MPs incubated with KOH solution.	Laser: Nd:YAG 213 nmSpot size: 100 μmICP-MS: QuadrupoleIsotopes: ^57^Fe, ^63^Cu, ^64^Zn, ^75^As, ^111^Cd, ^118^Sn, ^121^Sb, ^208^Pb, ^238^U	FTIR	- Depth profiling to investigated elemental distribution- Discrimination between adsorbed trace elements and additives- Quantitative analysis	81
Pristine MPs: PSM: soilS: —	Pristine MPs: PS, 3 μm, spheres	Pristine MPs mixed with soils.	Laser: ArF Excimer 193 nmSpot size: 100 μmICP-MS: QuadrupoleIsotopes: ^12^C	ICP-MS	- Non-destructive sampling with LA- SP LA-ICP-MS analysis- Advanced data processing	82
Pristine MPs: PA, PE, PET, PP, PVCM: freshwater/wastewater agedS: epoxy resin	Pristine MPs PE: 116.3 ± 68.0 μmPristine MPs PP: 357.0 ± 46.7 μmPristine MPs PA: 455.0 ± 49.1 μmPristine MPs PET: 23.1 ± 7.2 μmPristine MPs PVC: 157.4 ± 127.7 μmAll pristine MPs: fragments	Pristine plastics were grounded in a centrifugal mill to prepare MPs, part of MPs treated with freshwater/wastewater, filtered. Samples were mounted in acrylic resin and cross-sections were prepared.	Laser: ArF Excimer 193 nmSpot size: 7 μmICP-MS: QuadrupoleIsotopes: ^13^C, ^27^Al, ^48^Ti, ^59^Co, ^64^Zn, ^112^Cd, ^121^Sb, ^138^Ba, ^208^Pb	Optical microscopySEMLaser diffraction analysisLIBSICP-MSRaman spectroscopy	- Imaging of the elemental distribution of MPs with developed biofilm	22
Pristine MPs: PS, PMMA, PVCM: —S: dried 10 μL drops on PVDF membrane or glass microfiber filters	Pristine MPs: PS:2.07 ± 0.15, 3.10 ± 0.03, 3.97 ± 0.06, 5.19 ± 0.51, 6.05 ± 0.10, 8.12 ± 0.12, 9.87 ± 0.13, 20.15 ± 1.67 μmPristine MPs: PMMA:2.96 ± 0.09, 5.25 ± 0.25, 6.20 ± 0.20, 7.52 ± 0.12, 10.22 ± 0.30, 20.80 ± 1.76 μmPristine MPs: PVC: 2.62 ± 0.18, 3.69 ± 0.29, 5.59 ± 0.51 μmAll pristine MPs: spheres	MPs suspended in deionized water.	Laser: ArF Excimer 193 nmSpot size: varyingICP-MS: QuadrupoleIsotopes: ^13^C	ICP-MS	- Non-destructive sampling with LA- SP LA-ICP-MS analysis- Comparison of SP-ICP-MS and LA-SP-ICP-MS	83
Pristine MPs: PP, PET, PC, PS, PTFE, PVCM: —S: acrylic resin	Pristine MPs ∼ 5–270 μm, fragments	Pristine plastics were grounded in a centrifugal mill to prepare MPs, MPs embedded to silicon wafers with acrylic resin, cross-sectioned by manual polishing.	Laser: ArF Excimer 193 nmSpot size: 10 μmICP-MS: QuadrupoleIsotopes: ^13^C, ^46^Ti, ^65^Cu, ^115^Sn, ^123^Sb, ^208^Pb	LIBSICP-MSOptical microscopy	- Simultaneous LIBS/LA-ICP-MS- Characterization of MPs- Quantitative analysis of elements in MPs (laterally resolved)	29
Pristine MPs: PS, PVC, PMMAM: —S: silicon wafer	Pristine MPs: PS:2.07, 3.10, 4.78 μmPristine MPs: PMMA:2.96 μmPristine MPs: PVC: 5.0 μmAll pristine MPs: spheres	MPs suspended in deionized water and ethanol and transferred to high purity silicon wafer.	Laser: ArF Excimer 193 nmSpot size: varyingICP-MS: QuadrupoleIsotopes: 13C	ICP-MS	- Non-destructive sampling with LA- Size determination of individual MPs	84

**Nanoplastics**						
Pristine NPs: PSM: cucumber plantS: glass slide	Pristine NPs: PS: 823 ± 16 nm, 301 ± 11 nm, spheres, Eu-labeled	Plants were exposed to Eu-PS NPs, then the roots, stems, and leaves were slice thinly, cryo-cuts were applied onto glass slides.	Laser: Nd:YAG 266 nmSpot size: 50 μmICP-MS: QuadrupoleIsotopes: ^13^C, ^66^Zn, ^63^Cu, ^55^Mn, ^153^Eu	DLSSEMICP-MSFluorescence spectrometry	- Investigate the distribution of PS NPs in plants- Spatially resolved analysis	89
Pristine NPs: PSM: zebrafishS: quartz glass plate	Pristine NPs: PS: 100 nm, spheres, Eu-labeled	Fishes were exposed to Eu-PS NPs, separately or in presence Cd^2+^, then anesthetized, washed, fixed in 4% formaldehyde and places on the glass plate.	Laser: ArF Excimer 193 nmSpot size: 10 μmICP-MS: Triple-quadrupoleIsotopes: ^13^C, ^153^Eu, ^66^Zn, ^111^Cd	ICP-MSDLS	- Investigate the distribution of PS NPs in fish- Spatially resolved analysis	94

a — Data not presented.

##### 5.2.4.1. Spatially resolved investigations

###### 5.2.4.1.1 Direct depth-profiling

Depending on the size and shape of the MPs under investigation, LA-ICP-MS analysis may be possible in some cases without any sample preparation. This eliminates the risk of changing the composition during sample preparation due to contaminations or leaching. This approach was applied in the work of El Hadri *et al.* (2020).^81^ This work directly analysed the trace metal content of different MPs using depth profiling (depth resolution of 50 μm). With this approach, two different types of profiles were found. Some elements were present as additives with a constant distribution, while others were only found on surfaces near areas. The authors conclude that those elements are absorbed on the particles' surface or diffused into the particle during the weathering in the environment.

**Fig. 3:**
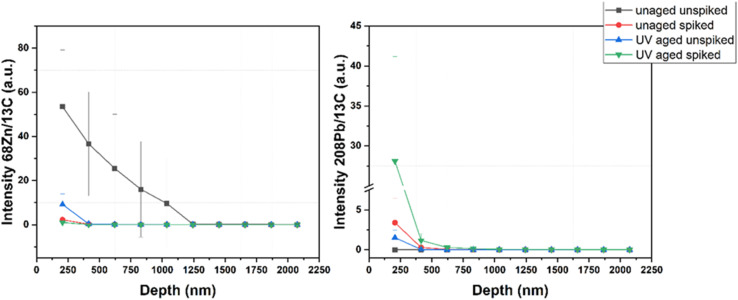
LA-ICP-MS depth profiling of artificially aged PS spheres.

**Unpublished results from the authors**: LA-ICP-MS depth profiling can also be applied with a higher depth resolution (∼200 nm) to analyse the metal content on surface near areas directly. This approach was applied to study the uptake of heavy metals and the release of inorganic additives based on artificial aging conditions. Therefore, PS spheres were exposed to UV radiation in artificial seawater spiked with 5 μg g^−1^ of Pb for 28 days. LA-ICP-MS depth profiling revealed an uptake of Pb in the first 500–750 nm of the MPs, which significantly increased when the sample was additionally exposed to UV radiation. Furthermore, Zn present as an additive in the first μm of the pristine particles is significantly leached from the particles into the environment during artificial sample aging (Fig. 3). This application highlights the possibility of using LA-ICP-MS depth profiling with a sub μm depth resolution to study the release and uptake of different metals in MPs, which is a crucial aspect when evaluating the impact of MPs on the environment.

###### 5.2.4.1.2 Imaging cross-sections

**Mounting particles and imaging cross-sections**: To overcome challenges associated with the irregular shape of MPs, mounting the particles under investigation and preparing cross-sections is reported in the literature. In this case, the MPs are embedded in a resin, and grinding prepares cross-sections. With this approach both information about the elemental content of the surface near areas and the bulk of the particle can be obtained.

**Fig. 4:**
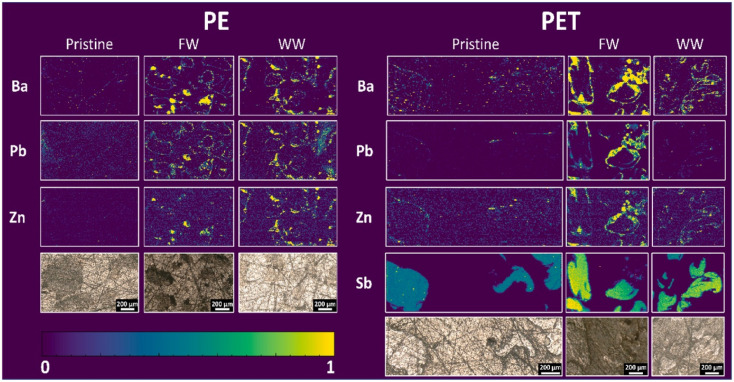
LA-ICP-MS imaging of cross-sections of pristine and aged PE and PET MPs in freshwater (FW), and wastewater (WW), revealing the lateral distribution of Ba, Pb, Zn, and Sb. Reproduced from ref. 22 with permission from Elsevier copyright [2025].

Even though this sample preparation approach provides a flat and well-defined surface suitable for LA-ICP-MS analysis, it should be mentioned that this strategy comes with the risk of altering the sample. Generally, every sample preparation step may introduce contamination or change the sample's composition. With this approach, the elemental distribution within individual particles can be mapped with a resolution in the low μm range. Besides getting insights into the elemental content of particles themselves, it is also possible to detect, *e.g.*, biofilm formation on the particles' surface by analysing biogenic elements. Additionally, the polymer type of individual MPs may be distinguished by marker elements characteristic of a specific polymer type. This was demonstrated in the work of Pořízka *et al.* (2023)^22^ where PP was identified based on an elevated ^27^Al signal, PVC was identified based on an elevated ^64^Zn signal, and PET was identified based on elevated ^121^Sb signal (Fig. 4). This work also applied LA-ICP-MS imaging to characterize the biofilm formation on the particles' surface. Differences in the elemental content of the biofilm based on different aging procedures (freshwater, wastewater) were found.^22^

In the work of Brunnbauer *et al.* (2023)^29^ the mounting approach was also used to image MPs with a combined LIBS/LA-ICP-MS setup. In this case, the problem with the need for matrix-matched standards for quantitative LA-ICP-MS results was tackled by combining LA-ICP-MS data with LIBS data. In this case, LIBS data was used to identify the polymer type of individual particles. With this information, the authors could apply matrix-matched standards to accurately quantify the metal content of both inorganic additives and trace constituents of an individual MP particle within the sample.^29^

**Imaging cryosections**: Wang *et al.* (2023)^89^ used LA-ICP-MS imaging to reveal the distribution of NPs in plants. Europium-doped PS NPs were used. Using a labelling approach instead of monitoring ^13^C allows for the use of the outstanding sensitivity of LA-ICP-MS for detecting metals to monitor NPs. Plants were cultivated in an environment with two different levels of NPs. Additionally, the authors investigated the stability of the Eu-doped PS spheres since leaching of the labelling regent could lead to biased results.^89^

###### 5.2.4.1.3 Imaging whole organisms

Very recently, europium-doped PS NPs have been visualized in biotic tissues, specifically in whole zebrafish specimens.^94^ These NPs were shown to bioaccumulate predominantly in the heart, head, liver, and pancreas of larvae when the fish were exposed solely to Eu-PS NPs. In contrast, when Eu-PS NPs and Cd^2+^ were co-exposed, the bioaccumulation sites of Eu-PS NPs also exhibited the corresponding bioaccumulation of Cd. Some NPs have a strong affinity for pollutant adsorption, making them carriers for pollutant transport and influencing their subsequent bioavailability. This phenomenon, known as the “Trojan-horse” effect, describes the potential role of NPs as carriers for chemicals, facilitating their uptake into organisms and leading to an increased toxicity.^94^

##### 5.2.4.2. Non-destructive LA-single particle (sp)-ICP-MS

The application of single-particle ICP-MS has attracted much attention in recent years. The introduction of ICP-ToF-MS instruments and further development in ICP-Q-MS instruments enable monitoring signals with a high time resolution. Therefore, detecting individual particles entering the plasma producing a short transient signal (200–500 μs) becomes feasible. This approach is most frequently applied for the analysis of inorganic nanoparticles^95^ but can also be used to detect and characterize MPs in liquids.^96,97^

Recently, a novel sampling approach has been introduced to analyse MPs with LA-ICP-MS. In this case, a low laser energy is used to desorb the particle from a substrate and transports it to the ICP-MS intact. An intact MP particle reaching the ICP-MS is typically detected as a very short transient signal of ^13^C (200–500 μs). This contrasts with conventional LA where a laser ablation process is used to ablate the particle and form a sample aerosol which creates a longer transient signal (5–10 ms).

This was reported for the first time by Lockwood *et al.* (2021)^82^ and further investigated by van Acker *et al.* (2023).^83^ These works demonstrated that the individual MPs reached the ICP-MS intact and that the obtained ^13^C signal showed a linear relationship with the particle size. Additionally, the authors showed that the ^13^C signal corresponded to the carbon content of the polymer type by analysing PS, PMMA, and PVC particles of different sizes (2–20 μm). Besides analysing MPs on a glass substrate, the authors also demonstrated that the approach worked to sample MPs from a filter material, enabling the application of real-life samples.

In the work of Brunnbauer *et al.* (2025)^84^ a novel calibration strategy was presented for the sizing of individual MPs based on the non-destructive sampling using LA. An in-house prepared PS thin film is used to ablate and introduce well-defined masses of carbon to the ICP-MS which can be used as a standard. With this approach, the size of PS, PMMA, and PVC particles in the range of 2–5 μm could be correctly determined.^84^

This approach provides unique advantages for MPs characterization. Besides determining the size of individual particles based on the ^13^C signal, modern ICP-TOF instruments allow the detection of the full elemental fingerprint of each particle. This could provide unique insights into processes related to aging, leaching, and uptake of various elements in different MPs. Additionally, the spatial distribution of MPs on a substrate is easily accessible.

### 5.3. Environmental implications

There are several ways to employ LIBS and LA-ICP-MS in the MPs analysis for pristine, artificially prepared as well as for environmentally relevant MPs. In the case of LIBS, the following uses have been already described (i) it was possible to distinguish MPs among various non-plastic (natural) particles',^12^ (ii) different MPs type were identified by using the PCA multivariate data analysis,^12^ (iii) the contamination of heavy metals in MPs was determined along with the places of their predominant sorption,^59^ (iv) the detection of trace element contents was performed,^61^ (v) the direct analysis of MPs with a developed biofilm after different aging processes was enabled directly without removing any biofilm,^22^ (vi) the analysis of MPs in soft tissues was done after their alkaline digestion,^64^ and (vii) the unique spatially-resolved fluorine detection in MPs (PTFE) was achieved.^29^

Nonetheless, LIBS is most frequently utilized in the metal contamination detection. The adsorption of potentially hazardous heavy metals on weathered MPs surface has a great significance, as MPs together with heavy metal contamination are a major environmental threat.^98^ This could be even augmented due to the possibility of a long-distance transport of MPs with adsorbed heavy metals to remote locations. Compared to large plastic items, MPs have a large specific surface area, high hydrophobicity, and a high tendency to interact with microorganisms, making them capable of surface adsorbing of heavy metals. For example, Al, Hg, Cd, Pb, Cr, Mn, Fe, Cu, Zn, or Cr^12,57,58^ have been recently detected in MPs collected in the environment as well as in MPs treated with metal solutions under the controlled laboratory conditions to evaluate adsorption mechanisms.^57,60^ In Vaisakh *et al.* (2023),^61^ the detected surface-adsorbed trace elements in MPs included heavy metals such as Al, Zn, Cu, Ni, Mn, Cr, and also other elements counting Na, Mg, Ca, Li, the achieved sensitivity is usually down to 10 ppm.^61^ These detection limits (LoDs) are typical also for another MPs LIBS studies, *e.g.*, 20 ppm for Cd/Pb.^57^ However, LA-ICP-MS offers a better sensitivity for a heavy metal/element contaminants direct detection in MPs as it will be discussed in the next paragraphs. Despite this fact, LIBS can still be used as a fast-screening tool for a huge number of samples without the risk of the introduction of impurities into a very sensitive measurement system, and also for distinguishing of MPs type in the same analysis based on obtained emission spectra.

During MPs lifetime in the environment and as they age, MPs interact with various pollutants. These interactions often lead to the adsorption of metals, organic compounds and nutrients.^43–45^ Conventionally, the presence of organic compounds associated with MPs is detected after desorption^99^ or in the case of metals, by either desorption or particle digestion techniques.^100–102^ However, the digestion of the particles reveals only bulk information and does not provide information about the location of metals—whether within biofilms, on the particle surface or due to migration in the bulk of the particles. However, LA-ICP-MS and LIBS allow to investigate the presence of metals and localize them on the particle.^99^ These findings are crucial for understanding not only the surface changes, but also the internal transformations that MPs undergo during aging processes in the environment.

Another important advantage for both techniques is the possibility of an accurate analysis of aged MPs directly without the need of biofilm removing priory the analysis. In a typical analysis, this biofilm usually needs to be removed. Methods including acidic and alkaline techniques, enzymatic, and oxidative treatments are widely used for this purpose, but these can lead to the loss or destruction of the particles or alter their surface properties,^103–105^ making subsequent analyses more difficult. Both laser-based methods have shown very promising results in the characterization of aged MPs heavily covered with biofilm developed in surface water and wastewater without the digestion of the biofilm.^22^

**Fig. 5:**
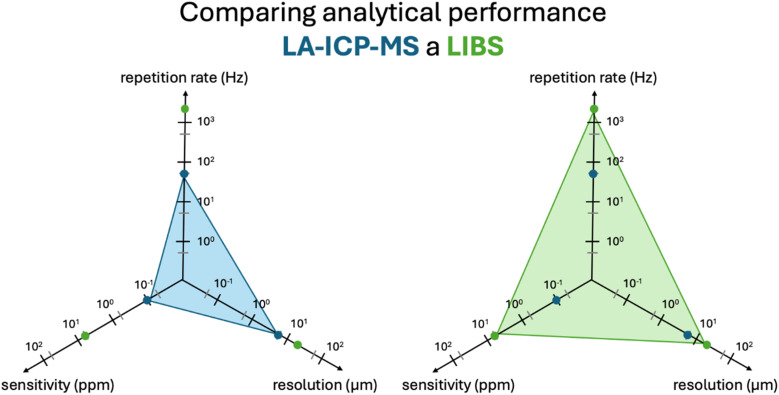
Comparison of LIBS (green) and LA-ICP-MS (blue) parameters in terms of speed of analysis (Hz), sensitivity (ppm) and spatial resolution (μm).

As described above, LA-ICP-MS typically excels with a better sensitivity compared to LIBS (Fig. 5) enabling not only the detection of lower concentrations but also a spatially resolved analysis with a better depth- and lateral resolution. This is beneficial to detect the trace metal profile of MPs as described in various work^22,29,81,89^ reporting detection limits in the single digit ppm range and below.

Besides the high sensitivity of LA-ICP-MS, the capabilities to obtain isotopic information must be highlighted: even though not reported in the literature yet, analysing the isotopic fingerprint of individual MPs, may provide information about the geographic origin which could help to better understand the fate of MPs in the environment.

Recent developments in the field of sp-ICP-MS have opened up new applications. With this approach large numbers of particles can be detected individually. Additionally, applying ICP-TOF-MS for detection allows to capture the full elemental fingerprint of each individual particle. Therefore, non-targeted elemental analysis becomes possible, which could be beneficial for further studies shining light on the complex interaction of MPs in the environment.

## 6. Conclusion and future perspectives

To sum up, the usefulness of LIBS/LA-ICP-MS analysis in MPs research papers published since 2020 is obvious. The utilization of both laser ablation-based techniques can be presented in tandem analysis, offering combined identification of polymer types with LIBS and high sensitivity metal detection with ICP-MS. Nevertheless, with this approach a compromise between laser parameters (wavelength, energy) has to be found limiting the resolution of the analysis to currently 30–50 μm. Separately, both techniques offer different benefits as it was discussed in detail for all research papers listed in this review. However, the detection of heavy metals and trace elements adsorbed onto MPs remain to be the crucial and the most suitable way. Nevertheless, even different previously discussed applications have their place in MPs analysis, especially the identification of MPs type simultaneously with the determination of various absorbed contaminants even in aged MPs covered with biofilm seems to be very promising.

From our point of view, the next steps in the LIBS/LA-ICP-MS utilization in environmental MPs analysis should lead to the direct detection of MPs in various abiotic and biotic matrices. Here, sample preparation is a crucial step. For abiotic matrices, *e.g.*, soils, ores, sands, and sediments, the collected samples could be prepared, as the most often way in the geological LIBS/LA-ICP-MS analysis, by simple pelleting by a hydraulic press.^106^ These pellets could be prepared directly from the collected samples after their proper mixing. However, usually a binder is also added to ensure a sufficient pellet strength. Nevertheless, the expected contamination by MPs of non-target soils or sediments should be low and the preconcentration of MPs will be necessary before the pelleting itself, done by simple sieving or by density separation in saturated aqueous salt solutions.^12,59^

In the case of analysis of biotic tissues, the situation differs significantly and several steps need to be taken. First, before the analysis of the organisms from the environment, the target organisms should be selected and exposed to MPs in well-defined laboratory toxicity tests. The used MPs need to be properly characterised and then artificially added in the exact amount to the surrounding testing media. After exposure, the organisms need to be prepared in a way that does not affect the MPs presence in or on organisms and then the optimization of the LIBS/LA-ICP-MS set-ups and measurement parameters has to be done. Second, various organisms collected directly from the environment (*e.g.*, aquatic or terrestrial organisms representing different trophic levels) require a specific approach of their correct sampling, sample transport, storage, and exact procedure for the preparation of the whole organisms or their specific parts. Finally, future experiments should lead to the determination of the MPs presence in humans, in their various soft tissues as organs or muscles, and to lead up to the utilization of LIBS/LA-ICP-MS in clinical research. In this case, the outcome situation is much trickier since the protocols for human tissues preparations and measurements need to be suitably prepared for the medical usage.

Although this review primarily focused on aquatic, sediment, and biological matrices, recent studies have increasingly highlighted the relevance of atmospheric and road dust as carriers of MPs and associated contaminants. These matrices represent important exposure pathways, particularly in urban and industrial environments, with growing evidence of long-range transport and inhalation risks.^107,108^ To date, no studies have applied LIBS or LA-ICP-MS for the detection or characterization of MPs in dust samples; however, both techniques are inherently well-suited for solid-phase analysis and could be highly valuable for this purpose. It is likely only a matter of time before these techniques are extended to this matrix, offering rapid, spatially resolved, and element-specific analysis of dust-bound MP particles. Future research should prioritize method development and validation in this promising but currently unexplored application area.

Besides, analysis of NPs is gaining more and more attention. Due to the high sensitivity, LA-ICP-MS has the potential for direct analysis of NPs. As discussed above, direct detection *via* Carbon may not be a feasible approach. Nevertheless, detection *via* inorganic marker elements (such as additives or contaminations) is promising. Uptake studies may also be carried out with *e.g.*: rare earth element-tagged NPs which can be detected with LA-ICP-MS with high sensitivity enabling detection of NPs. Feasibility of this approach was already demonstrated for single particle ICP-MS.^109^

## Abbreviations

AFM: Atomic Force Microscopy
CCD: Charge-Coupled Device
CF-LIBS: Calibration-Free Laser-Induced Breakdown Spectroscopy
CMOS: Complementary Metal-Oxide Semiconductor
DP: Double Pulse
EMCCD: Electron-Multiplying Charge-Coupled Device
FE-SEM: Field Emission Scanning Electron Microscopy
FLIM: Fluorescence Lifetime Imaging Microscopy
FTIR: Fourier-Transform Infrared Spectroscopy
ICCD: Intensified Charge-Coupled Device
ICP-MS: Inductively Coupled Plasma Mass Spectrometry
ICP-Q-MS: Inductively Coupled Plasma Quadrupole Mass Spectrometry
ICP-TOF-MS: Inductively Coupled Plasma Time-of-Flight Mass Spectrometry
LA-ICP-MS: Laser Ablation Inductively Coupled Plasma Mass Spectrometry
LC-MS/MS: Liquid Chromatography with Tandem Mass Spectrometry
LIBS: Laser-Induced Breakdown Spectroscopy
LoD: Limit of Detection
M: Matrix
MALDI-TOF MS: Matrix-Assisted Laser Desorption Ionization with Time-of-Flight Mass Spectrometry
MC-ICP-SF-MS: Multi-Collector Sector Field Inductively Coupled Plasma Mass Spectrometry
MPs: Microplastics
NPs: Nanoplastics
PCA: Principal Component Analysis
PADC: Polyallyl Diglycol Carbonate
PA: Polyamide
PBAT: Polybutylene Adipate Terephthalate
PC: Polycarbonate
PE: Polyethylene
PET: Polyethylene Terephthalate
PLA: Polylactic Acid
PLSR: Partial Least Squares Regression
PMMA: Polymethyl Methacrylate
PP: Polypropylene
PS: Polystyrene
PTFE: Polytetrafluoroethylene
PVC: Polyvinyl Chloride
Pyr-GC/MS: Pyrolysis Gas Chromatography Mass Spectrometry
S: Substrate
sCMOS: Scientific Complementary Metal-Oxide Semiconductor
SEM-EDS: Scanning Electron Microscopy with Energy Dispersive X-ray Spectroscopy
SEM-EDX: Scanning Electron Microscopy with Energy Dispersive X-ray Spectroscopy
SP: Single Pulse
SPR: Single-Pulse Response
TED-GC/MS: Thermal Extraction and Desorption Gas Chromatography Mass Spectrometry
XRF: X-ray Fluorescence

## Conflicts of interest

The authors claim on conflict of interest. It is original work that was submitted only as this manuscript.

## Data Availability

Data are available on demand.
